# EXPERIENCES OF SOCIAL ISOLATION BY MANDARIN AND PUNJABI-SPEAKING OLDER IMMIGRANTS LIVING IN CALGARY

**DOI:** 10.1093/geroni/igad104.3326

**Published:** 2023-12-21

**Authors:** Prince Ekoh, Change Zhu, Christine Walsh, Sepali Guruge

**Affiliations:** University of Calgary, Calgary, Alberta, Canada; University of Calgary, Calgary, Alberta, Canada; University of Calgary, Calgary, Alberta, Canada; Toronto Metropolitan University, Toronto, Ontario, Canada

## Abstract

Loneliness and social isolation have continued to be risk factors for the health and overall quality of life of older adults globally. The impact of loneliness on healthy ageing may even be more profound for older immigrants in Western countries like Canada, where there is a different language and culture from that of their home countries. As part of the Inclusive Communities for Older Immigrants (ICOI), which seeks to create knowledge about social isolation and improve social connectedness among older immigrants across Canada, we examined the experiences of social isolation amongst older Mandarin and Punjabi-speaking immigrants aged 60 years and over living in Calgary. Quantitative data was collected from 64 (33 Mandarin-speaking and 31 Punjabi-speaking) older adults in Calgary, Alberta. The data were cleaned and analysed using descriptive analysis with the aid of Statistical Packages for Social Sciences (SPSS), and findings showed that older immigrants conceptualised social isolation as limited participation in various community activities. Our results also showed that older immigrants perceived physical and mental health status, social conditions and environmental conditions as significant factors predisposing older immigrants to social isolation. The study, therefore, recommends designing interventions that will increase older immigrants’ participation in community activities considering their age, language, disabilities, health, and social conditions.

